# Linking hosts, landscapes, and climate to advance zoonotic arbovirus forecasting

**DOI:** 10.1038/s41598-026-46902-2

**Published:** 2026-04-06

**Authors:** V. A. Akshay, J. Alex Baecher, Nathan Burkett-Cadena, James T. Thorson, Yesenia Sánchez, Yasmin Tavares, Amely Bauer, Robert Guralnick, Lindsay Campbell

**Affiliations:** 1 School of Natural Resources and Environment, IFAS, University of Florida, Gainesville, FL 32611 USA; 2 Florida Natural History Museum, University of Florida, Gainesville, FL 32611 USA; 3 Florida Medical Entomology Laboratory, IFAS, University of Florida, Vero Beach, FL 32962 USA; 4 Department of Entomology and Nematology, IFAS, University of Florida, Gainesville, FL 32611 USA; 5 Resource Ecology and Fisheries Management, Alaska Fisheries Science Center, Seattle, WA 98115 USA; 6 Department of Wildlife Ecology and Conservation, IFAS, University of Florida, Gainesville, FL 32611 USA; 7 Center for Advanced Systems Understanding - CASUS, Helmholtz-Zentrum Dresden-Rossendorf, 02826 Görlitz, Germany; 8 Department of Community Ecology, Helmholtz Centre for Environmental Research - UFZ, 06120 Halle (Saale), Germany

## Abstract

Forecasting zoonotic mosquito-borne viruses remains a critical challenge because transmission depends on dynamic, multitrophic interactions among vectors, hosts, pathogens, and the environment. We integrate long-term sentinel chicken surveillance in Florida with environmental data to build a predictive framework for eastern equine encephalitis virus (EEEV), a zoonotic mosquito borne disease of concern to human and equine health. Models captured environmental drivers and latent spatiotemporal structure, achieving strong predictive accuracy. Models also revealed nonlinear effects of moderate precipitation a year prior to sampling, higher minimum temperature a month prior to sampling, and moderate and high forest and wetland cover on increased EEEV seroconversion. Retrospective predictions showed distributions of virus activity across regions, consistent with *Culiseta melanura* vector ecology. We also calculated associations between predicted EEEV seroconversion and abundance estimates for suspected avian hosts using eBird data. Seasonal shifts among migratory and resident birds with predicted virus activity for key species suggests spring migrants play a role in amplification, residents in summer persistence, and overwintering groups as potential reservoirs. These results demonstrate ecological forecasting is feasible at management-relevant scales, with broad potential to extend to other arbovirus systems. By integrating traditional surveillance with community science eBird data, our framework advances predictive capacity and ecological understanding of zoonotic arboviruses.

**Supplementary Information:**

The online version contains supplementary material available at 10.1038/s41598-026-46902-2.

## Introduction

Zoonotic mosquito-borne diseases pose a major global health threat, with capacity to rapidly spread to new areas^1^. Transmission in these systems is the result of multitrophic interactions among hosts, vectors, and pathogens, shaped by both intrinsic factors (e.g., demographics, vector competence) and extrinsic environmental conditions (e.g., climate, habitat, and biotic interactions)^2,3^. In vector-borne diseases (VBDs) maintained between avian hosts and mosquito vectors, host movement and migration, seasonal phenology of mosquito populations, and overlap of components in space and time determine enzootic pathogen maintenance, amplification, as well as spillover to humans and other animals^4^. These systems often include multiple hosts and vectors, and predicting transmission risks to humans or animals, such as horses, remains a wicked problem impacting disease prevention^5–7^. Rather than proactively assessing where risks might be highest, most disease prevention efforts are reactive, attempting to mitigate outbreaks already in progress^8,9^. Because disease system components are difficult to measure fully across space and time, developing predictive capacity requires intensive monitoring and modeling frameworks that can integrate and harmonize data from multiple sources, while capturing the spatiotemporal structure of unobserved processes driving virus activity^10^. Such an integrated approach allows incorporation of new data streams while maintaining the potential to scale prediction and forecasting across broader areas.

Eastern equine encephalitis virus (EEEV; *Alphavirus*, *Togaviridae*) is a zoonotic mosquito borne disease system maintained in an enzootic transmission cycle between the primary mosquito vector, *Culiseta melanura*, and multiple avian host species with occasional spillover transmission to humans and horses^11–15^. EEEV in humans is relatively uncommon, but consequences can be severe, including long lasting neurological problems, and EEEV has the highest mortality rate of any arbovirus in the U.S. at ~ 30% in those developing neuroinvasive disease^16,17^. The virus has a broad geographic distribution spanning eastern North America, the Caribbean, Central America, and portions of South America^18–20^ with some distributional overlap with closely related Madariaga virus in South and Central America, which primarily causes disease in equines^21^. In the northern part of its distribution, the virus continues to expand its range^13^, leading to its designation as an emerging infectious disease, as well as a select agent due to its potential use as a bioweapon^22^. Despite the growing frequency and intensity of EEEV outbreaks^13^, anticipating the distribution and dynamics of EEEV activity remains a persistent challenge, and the ability to proactively forecast risk is non-existent.

In Florida, EEEV is enzootic with seasonal spillover to equines, and occasional cases in humans and other animals, including emus^23^. Peninsular Florida, in particular, may impact EEEV transmission ecology more broadly, given that it is part of a major intercontinental migratory bird flyway with extensive stopover habitats, which could serve as an important source of virus dispersal^23,24^. To monitor for EEEV, the Florida Department of Health (FDOH), in partnership with mosquito control programs, maintains one of the longest-running arbovirus surveillance programs in the U.S., monitoring sentinel chickens across hundreds of coops statewide^25^. This spatially extensive, long-term dataset provides a data basis for integrating long-term surveillance with biotic and abiotic information to predict virus activity and transmission hazard. Building a more predictive framework will not only strengthen early warning systems, but also deepen fundamental insights into multitrophic disease dynamics and enhance ability to proactively forecast zoonotic vector-borne disease hazard.

Our approach in this work leverages new advances in spatiotemporal modeling that can capture both measured environmental correlations and spatiotemporal structure in unmeasured variables to improve model predictions compared to non-spatiotemporal models^26,27^. Beyond improved prediction, model outputs may reveal patterns that can be compared with complementary data streams, including community science observations (i.e. eBird), to correlate suspected host dynamics with patterns of virus activity. These factors are particularly relevant for EEEV where long- standing questions about avian host migration and overwintering dynamics in Florida and their connection to the broader spatiotemporal ecology of the system remain unresolved^23^.

Here we: 1) develop spatiotemporal predictive models of monthly EEEV sentinel chicken seroconversion representing virus activity across Florida between 2005 and 2019 and 2) examine correlations between estimated values of avian abundances from community science eBird data and predicted EEEV activity between 2005 and 2019 for 12 resident and migratory species that are suspected avian hosts. Although primarily interested in predicting virus activity with statistical tools, our multitrophic approach provides a step toward needed realism about host distributions and abundances, which are most often unmeasured, particularly at this scale. Here, we predict virus activity in space and time, as well as draw inference about the abiotic and biotic drivers underlying these patterns. We expect higher seroconversion rates at locations surrounded by greater forest and wetland areas, given known habitat preferences of *Culiseta melanura*, as well as with greater precipitation in earlier seasons, based on previous studies in the northeastern U.S. We also expect lower seroconversion rates with very high temperatures, which can slow mosquito activity and increase mortality, supported by our previous work on West Nile virus^10^. Finally, greater abundances of spring migratory avian hosts will precede elevated predictions of EEEV seroconversion by 2–3 months, while resident species will show peak correlations with predicted seroconversion during the breeding summer season. Fall migrants and overwintering avian species will show strong contemporaneous associations over the winter as possible reservoirs of the virus.

## Results

### Initial model results

**Fig. 1:**
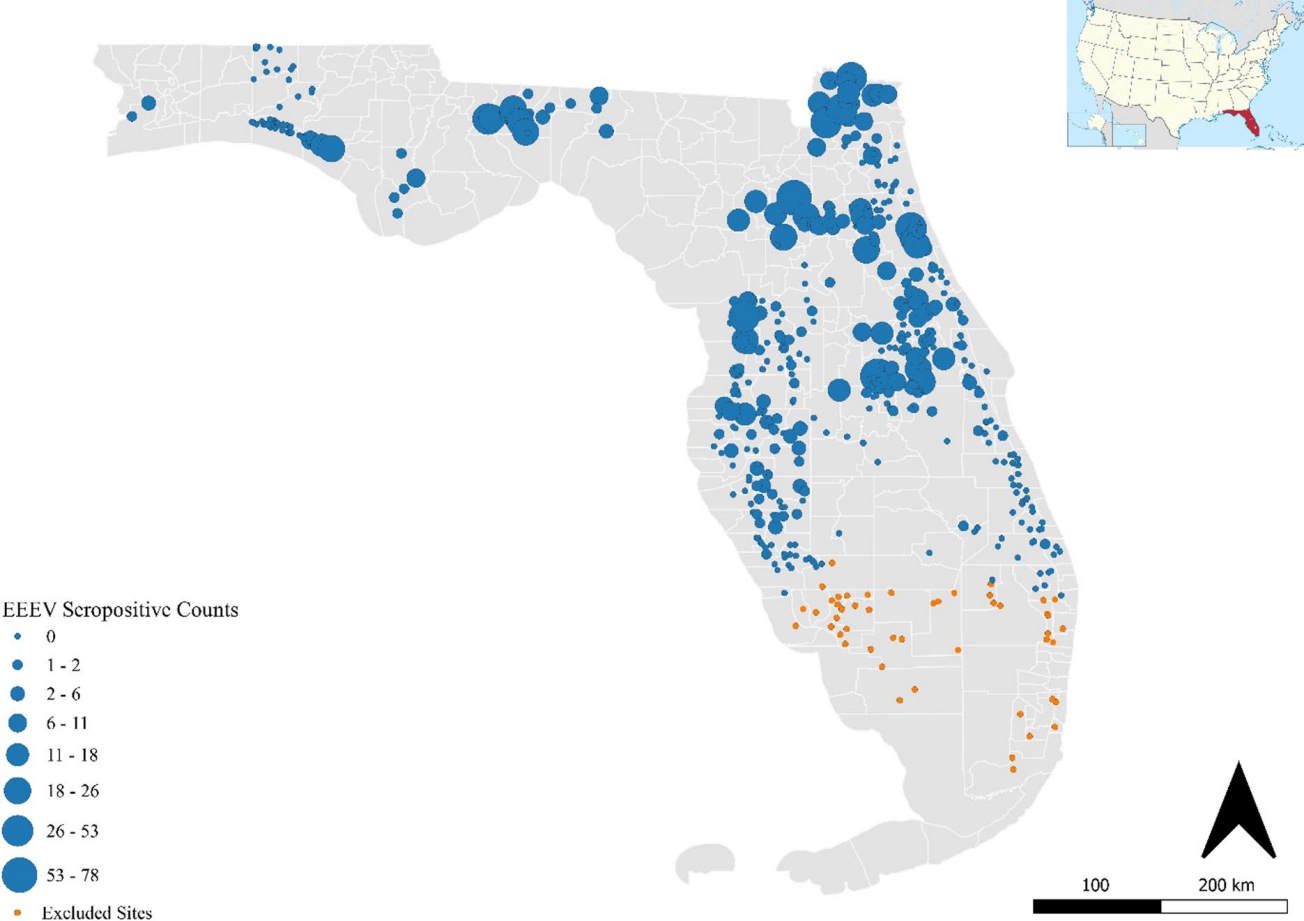
The spatial distribution and total counts of EEEV seroconversion across sentinel chicken coops from 2005 to 2019. Location of sentinel chicken coops are depicted by circles, sized by seropositive counts across the study period. We excluded coops in South Florida (orange circles) where EEEV was never recorded. Maps were created using R version 4.5.2 and QGIS version 3.26.3.

We compiled and analyzed Florida Department of Health EEEV sentinel chicken seroconversion surveillance data representing virus activity spanning 15 years, developed monthly spatiotemporal models to predict transmission hazard across Florida’s diverse landscapes and identified associations between estimated avian abundances and phenology and predicted virus activity (Fig. 1). We start first with our abiotic-focused models. After systematically removing collinear variables evaluated using variance inflation factors (VIF > 5), and performing a stepwise backward selection, our final model incorporated 8 key abiotic predictors: lagged cumulative precipitation (1, 5, and 12 months), lagged maximum temperature (6 and 12 months), minimum temperature (1 month), and percentage forest and wetland land cover (Table 1).

**Table 1:** Description of the predictor variables used to model spatiotemporal EEEV seroconversion proportions in the state of Florida (USA).

Effect	Term	Description	Variable type	Parameterization
Fixed	*prcp_lag1*	Cumulative precipitation (1 mo. lag)	Continuous	2nd order orthogonal polynomial
	*prcp_lag5*	Cumulative precipitation (5 mo. lag)	Continuous	2nd order orthogonal polynomial
	*prcp_lag12*	Cumulative precipitation (12 mo. lag)	Continuous	2nd order orthogonal polynomial
	*tmax_lag6*	Max temperature (6 mo. lag)	Continuous	2nd order orthogonal polynomial
	*tmax_lag12*	Max temperature (12 mo. lag)	Continuous	2nd order orthogonal polynomial
	*tmin_lag1*	Min temperature (1 mo. lag)	Continuous	2nd order orthogonal polynomial
	*Forest*	Proportion forest cover	Continuous	2nd order orthogonal polynomial
	*Wetlands*	Proportion wetlands	Continuous	2nd order orthogonal polynomial
Random	*County*	County name	39-level factor	Intercept nested above site_id
	*site_id*	Monitoring site name	476-level factor	Intercept nested below county

Terms included in this table result from a model selection and variable reduction procedure using spatial generalized linear mixed effects models (GLMMs) prior to fitting full spatiotemporal models (see methods). Descriptions and parameterization details are provided for each variable.

Next, we implemented a spatiotemporal framework incorporating Gaussian Markov Random Fields (GMRFs), which substantially outperformed all alternative model formulations. This model showed strong temporal autocorrelation (AR1 ρ = 0.73), with spatial correlation extending to 82.28 km (Matérn range), and substantial spatiotemporal variability (marginal SD = 1.75), indicating spatiotemporal structure in EEEV seroprevalence beyond that explained by covariates. The best GMRF model explained 37.7% of deviance with an AIC of 6164.1, compared to 29.9% and an AIC of 6920.7 for the best performing non-spatial model (Table S1.1).

### Model validation

**Fig. 2:**
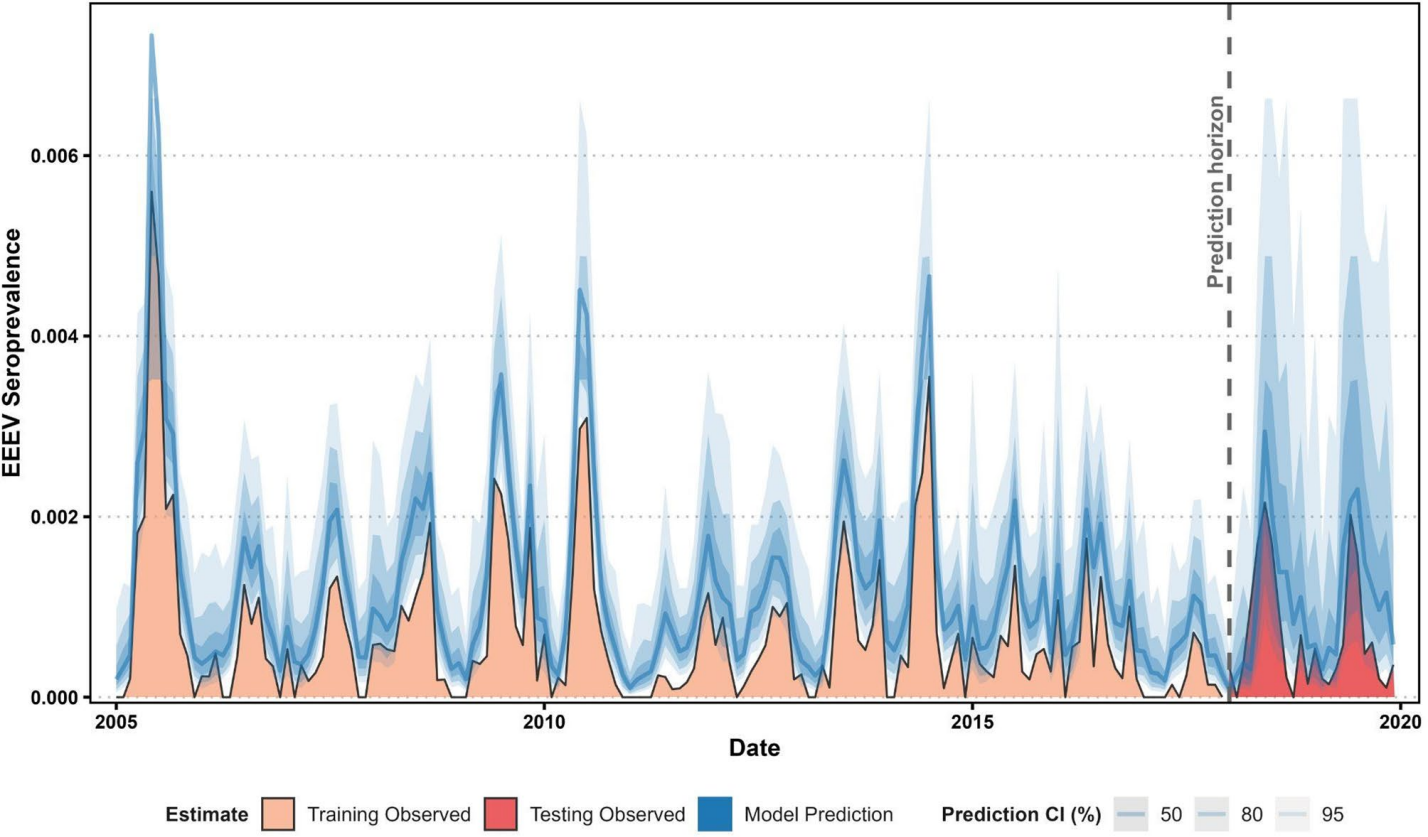
Temporal trends of EEEV seroprevalence in Florida (2005–2019) from empirical data aggregated at the monthly level (orange = training, red = testing) and model predictions (blue). The model was trained on 2005–2017 data and evaluated using 2018–2019 out-of-sample predictions (dotted vertical line). Model predictions are presented as point estimates (blue line) and 50, 80, 90, and 95% confidence intervals (opaque blue ribbons).

We withheld the last 2 years of surveillance data (2018–2019) for model validation. That validation showed strong predictive performance accurately capturing the magnitude and timing of EEEV activity (overall RMSE = 0.007457; Table S1.2) in both out years, but in general, overestimates exact seroprevalence values. Errors remained low across months (1.88 × 10⁻5 to 0.015352), with predictions consistently within empirical confidence intervals across most months (Fig. 2). Performance was slightly better in 2019 (RMSE = 0.007241) than in 2018 (0.007668), but both out years demonstrated potential for operational forecasting.

### Environmental determinants of and spatiotemporal trends in EEEV activity

**Fig. 3:**
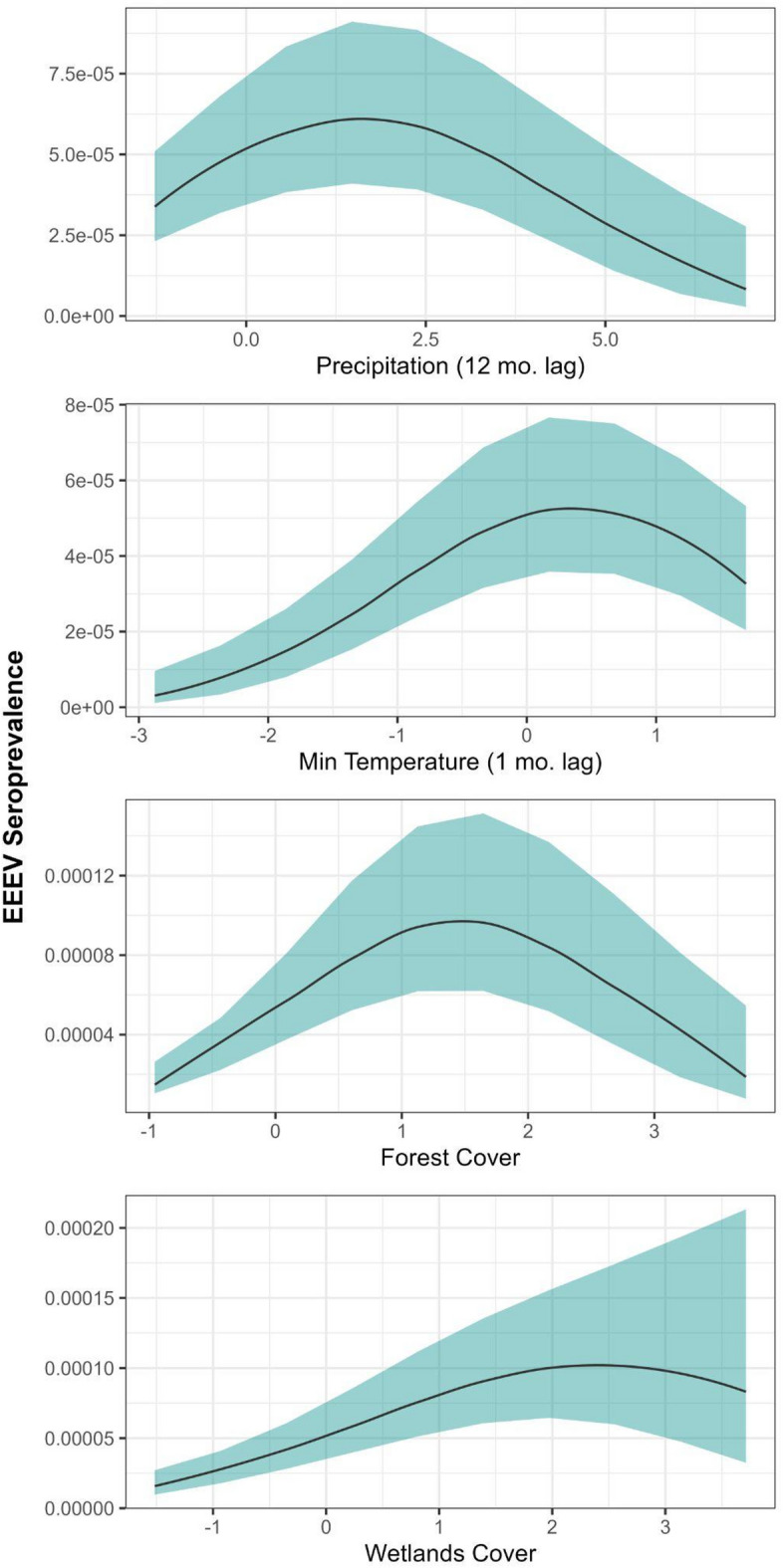
Marginal response plots of predicted Eastern Equine Encephalitis Virus (EEEV) seroprevalence from a spatiotemporal model of Florida sentinel chicken surveillance (2005–2019) aggregated at the monthly-level. Environmental covariates were measured contemporaneously or with 1-, 5-, 6-, or 12-month lags, as indicated by variable names.

Environmental response curves (Fig. 3) revealed complex, non-linear relationships with virus activity. The model identified strong precipitation effects, with intermediate prior-year rainfall (12-month lag) predicting elevated seroconversion. Moderate forest cover was the strongest landscape predictor, showing positive effects that decreased at higher coverage, while wetlands also had strong positive associations and plateaued at highest coverage, which are both consistent with known EEEV vector and host ecology^13,28^. For the full set of predictors and parameter estimates see Table S1.3. Parallel analyses conducted on the second candidate model set yielded qualitatively similar results, with detailed results provided in Supplementary Materials (Table S1.4).

**Fig. 4:**
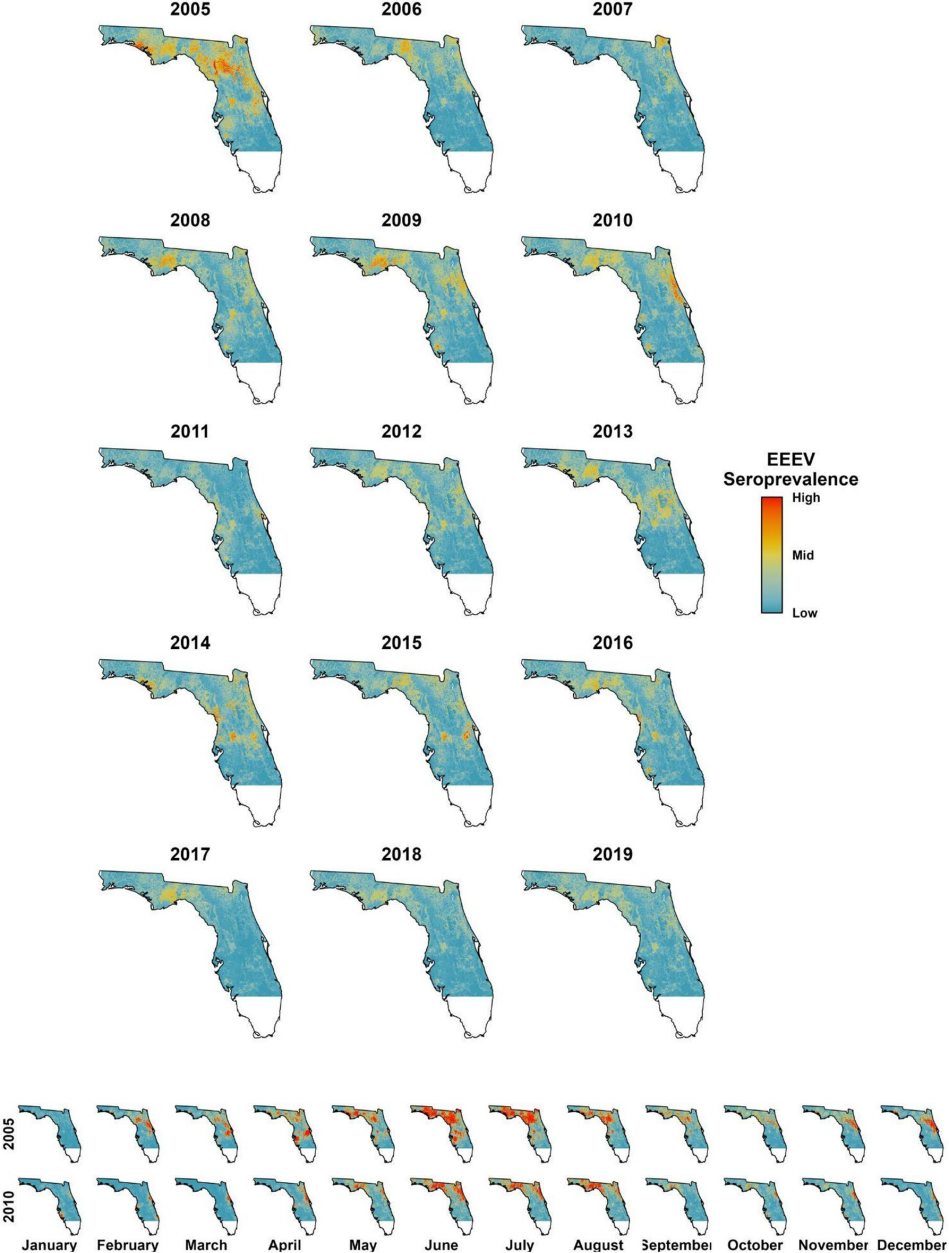
Statewide predictions of EEEV seroprevalence from Florida sentinel chicken surveillance system aggregated to year from 2005 to 2019 and monthly predictions for 2005 and 2010. All predictions are provided with a quantile truncation for ease of visualization at the 98th percentile of values.

Statewide retrospective predictions revealed distinct temporal patterns in EEEV transmission dynamics over the study period in Florida (Fig. 4), with peak statewide activity in 2005, spanning the Panhandle, Gulf Coast, and north-central regions. Predicted virus activity declined through 2007 before increasing from 2008 to 2010. A second decline during 2011–2012 was followed by renewed increases from 2013 to 2015, with activity shifting toward central Florida. Monthly predictions provided finer resolution insights into seasonal patterns, revealing summer transmission peaks from June through August alongside persistent low-level activity year-round (Figure S1.1). The model’s highest predicted virus activity occurred during June 2005 across the Gulf Coast and extensive areas of central and north-central Florida. Prediction uncertainty was greatest in regions with sparse sentinel chicken surveillance coverage and mean seroprevalence was predicted highest along forested wetland areas across the Panhandle and northern Florida (Figure S1.2).

### Associations of viral activity with migratory and resident birds

**Fig. 5:**
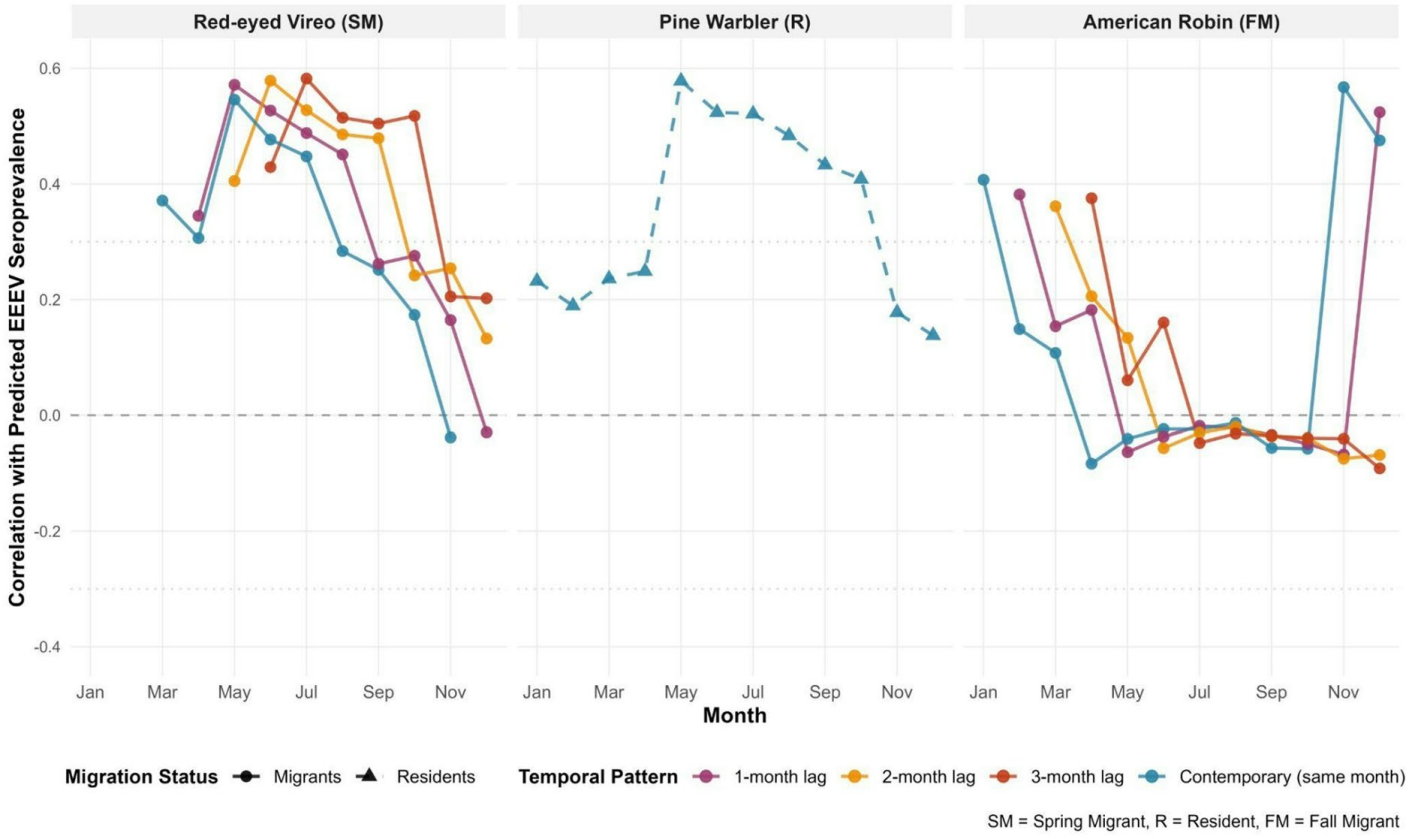
Monthly pixelwise Pearson correlations (y-axis) between predicted EEEV seroprevalence (2005–2019) and bird abundance across seasons (x-axis) for three species with the strongest associations (r ~ 0.60). Monthly predicted seroconversion was aggregated to the 3-km^2^ resolution eBird Status and Trends data and then averaged across years, and the eBird Status and Trends data provided monthly species-specific relative abundance derived across multiple years to represent a “typical year”. For resident species, we assessed contemporaneous correlations; for migratory species, we also calculated 1–3 month lags to observe potential delays in virus amplification. Correlations for all 12 species are shown in Figure S1.3.

We also analyzed correlations between eBird Status and Trends data and predicted EEEV across the state. The eBird Status and Trends data provides a weekly relative abundance estimate derived across multiple years for a single “typical year” value for each species. Here, we aggregated the weekly temporal resolution to monthly to align with our EEEV model. In addition, we derived a single monthly EEEV value for each pixel across the study area by aggregating predictions to the 3-km^2^ resolution of the eBird data and averaging predicted seroprevalence values across the study period. Together these steps allowed us to observe baseline correlations between the monthly timing and estimated abundances of avian hosts and predicted virus activity for a “typical year.” Associational analyses between predicted seroconversion rates and abundances of suspected avian hosts revealed marked seasonal patterns across the avian community, in directions expected by migratory status. Spring migrants showed particularly strong associations with predictions of elevated transmission hazard, led by the Red-eyed Vireo (r = 0.58, July across a 3-month lag, Fig. 5). Year-round residents exhibited varied responses, with Pine Warbler showing peak associations with EEEV seropositive rates during spring breeding season (r = 0.58, May) while Northern Cardinal peaked during summer months (r = 0.36, July). Common Yellowthroat, another resident species, demonstrated moderate spring associations (r = 0.38, May), suggesting shared seasonal patterns among certain resident taxa (Figure S1.3). Winter migrants presented a more complex picture, with American Robin showing strong late-season correlations (r = 0.57, November) and Yellow-rumped Warbler peaking in December (r = 0.45). Notably, several species exhibited negative or very weak correlations with predicted virus activity, including Green Heron (r = -0.37, August), Black-crowned Night Heron (r = -0.31, July), and Wood Thrush (r = -0.12, December across a 3-month lag).

## Discussion

Here we demonstrate the capacity to forecast near-term EEEV activity across a regional scale, while more fully accounting for abiotic and biotic factors underlying transmission dynamics. This step forward is enabled by integrating four separate key elements: (1) A broadly sampled multi-site, multi-year time-series of EEEV activity measured across most of Florida; (2) A set of statistical models that can appropriately account for spatiotemporal structure in unmeasured variables, thus providing needed information about lag effects that underlie effective near term forecasting; (3) Knowledge of the habitat preferences of the main vector species that transmit EEEV that can be incorporated into models; 4) Knowledge of the key bird species that likely serve as EEEV hosts, whose relative abundances are now available at scale through harmonized citizen science data collected by an avid volunteer birding community. We bring all these resources and advancements into one framework that showcases underlying processes and the capacity to build operational forecasts of transmission hazard, and we establish a foundation to further examine both migratory and resident host species in disease dynamics across this broadscale system.

*Landscape and climate determinants of viral activity:* As expected, moderate forest cover and extensive wetlands were strongly associated with elevated seroconversion, consistent with the known hardwood swamp habitat of *Culiseta melanura* vectors^28^ as well as suspected bridge vectors, including *Coquillitidia perturbans* which is found widely in freshwater marsh habitats^29^, *Culex erraticus* and *Aedes vexans*, which are distributed across a broad range of wetland habitats^30–32^, and salt marsh mosquitoes, such as *Aedes sollicitans* and *Aedes salinarius*^31,33^ aligning with patterns reported for equine cases and other landscape studies in Florida and Connecticut^28,31,33–35^. This finding further underscores forest and wetland as important habitats in the EEEV system, linking mosquito habitat preferences with virus activity. As well, these findings have potential implications for human exposure through ongoing development, as well as restoration efforts, which have been suggested in EEEV emergence in the northeastern U.S.^36^. Consistent findings of these landscape predictors across disparate regions highlights their potential to scale beyond regional study areas and generalize across the full geographic range of EEEV. This finding is in sharp contrast to West Nile virus, another mosquito vector and avian host system, where although multiple landscape associations have been identified in individual regions^37–39^, consistent landscape predictors across Florida and broader areas have remained elusive^10,35,40,41^. This work points to the potential for targeted mosquito abatement and control activities near forest and wetland habitats as a means to reduce transmission hazard in these areas.

We also found elevated seroconversion was associated with mild minimum temperatures 1 month prior to sampling and intermediate precipitation levels 12 months prior to sampling. Warmer minimum temperatures in the time period immediately preceding seroconversion likely promotes increased mosquito survival and activity, which may sustain virus circulation and enhance transmission in the active season^3,42^ because mosquito development, metabolism, and viral replication all accelerate with warmer, but not extreme, temperatures. Intermediate levels of precipitation 12 months prior to sampling can also impact mosquito abundance and survival needed to maintain virus circulation during the following winter or spring months. Although^28^ found that extremely wet hydrological conditions 8 months prior to the EEEV season was associated with a greater number of positive mosquito pools in the northeastern U.S., extreme precipitation can also result in larval and egg flushing of mosquitoes^43^ and too dry conditions can reduce overall habitat availability, and impact mosquito activity, survival, and abundances^44^.

Overall, climate variables at distinct time scales, combined with landscape data, offer complementary pathways for anticipating and ultimately forecasting elevated EEEV activity. We show here that predictions for out years generally match the magnitude and timing of EEEV activity for hold out data. Of particular note is the ability of these models to effectively predict EEEV based on calibration and validation data, despite a relatively low seroconversion rate. A 12-month precipitation lag provides long lead times to guide strategic planning and resource allocation, while contemporaneous minimum temperature aligns with the short-term horizons of existing climate forecasts to refine local response. Together, these predictors bridge seasonal to near-real-time scales, creating a foundation for operational early-warning systems that can target surveillance and control across hazardous landscapes.

### Retrospective predictions

Retrospective monthly predictions and annual summaries of EEEV activity provide a comprehensive view across the state and provided the first tool to observe dynamic distributions of this system in this region. A key time period of elevated EEEV activity was in 2005 when 50 counties reported virus activity, including 5 human cases and 3 deaths^45^. Our models predicted elevated EEEV activity in June, July, and August 2005 in Gadsden and Leon Counties in the Florida Panhandle, in Suwannee County in north central Florida, as well as Pasco County in central Florida, where human cases occurred in July and August^45^. Additional retrospective predictions demonstrate variation in monthly spatiotemporal dynamics of virus activity across the state (Figure S1.1). Outputs have the potential to provide new insights into the ecology of disease system dynamics, including the role of early season transmission in later season amplification and how predicted virus activity links to downstream human and equine transmission risk.

### A framework for integrating host dynamics and for process-oriented forecasts

A second element of our framework was integrating eBird community science data to explore associations between avian phenology and abundances and predicted EEEV seroconversion. The results reveal striking seasonal patterns that align with hypothesized host roles where spring migrants may introduce or amplify low-level circulation, resident species appear to sustain summer transmission, and overwintering migrants may maintain virus activity during cooler months^23^. Red-eyed Vireos, which arrive in March, and to a lesser extent early season Hermit Thrush, were both correlated with peak transmission in May–July, consistent with the possibility of low level early season amplification followed by amplification during the breeding season. Pine Warbler and other resident songbirds showed positive correlations only during the breeding season, supporting their suspected role as amplifying hosts. Strong correlations between American Robins and predicted EEEV activity in fall and winter were also consistent with their implication as important hosts^46^. Eastern Phoebe and Yellow-rumped Warbler showed weaker but similar patterns, suggesting a potential role in sustaining virus activity during the non-breeding season. By contrast, Wood Thrush showed unexpectedly weak correlations despite its prominence as a host of EEEV in northeastern systems^13,47^, and wading birds such as Green Heron and Black-crowned Night Heron exhibited weak negative correlations, raising the possibility of dilution effects, though their host competence remains unknown. Overall, the alignment between migration phenology, abundances, and correlation peaks show compelling patterns supporting avian movement in shaping EEEV landscape-scale dynamics. These species-specific patterns highlight the potential of integrating community science with traditional surveillance to better disentangle host contributions and strengthen early-warning systems for zoonotic arboviruses. Still, much needs to be done to better integrate bird abundance data into modeling frameworks, as we discuss below.

### Caveats, conclusions and future directions

While our models and predictions are robust and the most comprehensive to date, we also recognize some key limitations and caveats. First, historical records of EEEV sentinel chickens indicate whether susceptible chickens were placed in coops in a mosquito program for the sampling week but do not indicate the exact number of birds within each coop. The sentinel chicken surveillance program follows a standardized protocol with ~ 6 chickens tested weekly per coop. However, this number can vary slightly, which may introduce some uncertainty in our weighting term. The number of chickens tested in each coop is now recorded electronically, and future analyses will benefit from this more precise number.

A second key limitation that precluded a joint analysis of abiotic and biotic factors in the same predictive modeling framework was the nature of the eBird data we used. Briefly, although derived from multiple years, eBird abundance data was available for only a single “typical year”. This limitation means that we could not account for interannual variation in bird abundance, and while it may be possible to back calculate a proxy for this from raw eBird datasets, it was out of scope of the intended effort here. Despite this challenge, eBird data representing both migratory and resident species of suspected hosts provided insights into key associations between the timing and distribution of avian species abundances and predicted EEEV.

A next step is to expand this framework from retrospective predictions to forecasts of EEEV seroconversion and to incorporate eBird data and near-real-time and forecasted bird migration data from platforms such as BirdCast^48^, which could provide additional information toward improving early-warning capacity. More broadly, linking avian movement, abundances, and phenology with virus activity has the potential to create a foundation for scalable ecological forecasting of mosquito–bird vector-borne disease systems in Florida and beyond. Emerging data streams from community science and cross-sector monitoring can allow forecasts to capture macroscale drivers of host and vector dynamics, while informing local-scale hazard assessments under accelerating global change. Essential to this effort is continued integration of biological and environmental data with modeling approaches that account for unmeasured latent variables, thereby improving the ability to predict when and where elevated transmission hazard is most likely to occur.

## Materials and methods

### Study area

Florida spans subtropical to tropical climates, characterized by year-round warmth and pronounced wet (May to October) and dry (November to April) seasons. The state has experienced significant environmental change, including rising minimum temperatures, altered precipitation over the past 30 years^49^, and ongoing land use transformation, primarily from natural to agricultural habitats and urban development^50^. Land cover across the state is diverse, ranging from gradients of urban development to agricultural lands, as well as forests and wetlands, including hardwood swamp habitats, and salt marsh environments^50^. Additionally, the Florida peninsula is a critical stopover site for migratory bird species along the Atlantic flyway and also serves as an overwintering ground for numerous North American bird species^51^.

### Sentinel chicken data and preparation

We utilized EEEV surveillance data from the Florida Department of Health’s sentinel chicken monitoring program, which operates through partnerships with local mosquito control programs across the state^25^. Following standardized protocols, alphavirus-susceptible chickens are housed at monitoring sites and sampled weekly throughout large portions of the year^25^. Blood samples undergo initial screening with hemagglutination inhibition tests, followed by IgM enzyme-linked immunosorbent assays (ELISA) to identify antibody-positive samples. If ELISA results are negative or unequivocal, a Plaque Reduction Neutralization Test (PRNT) is performed to differentiate between EEEV and Highlands J virus.

Following the approach outlined in^10^, we created a bioinformatics pipeline to digitize and quality-control nearly two decades of surveillance records from 2001 to 2019. Paper reports were processed using Amazon Web Services Textract optical character recognition, then cleaned and formatted using R ‘tidyverse’ functions^52^ and OpenRefine software^53^. Our initial dataset comprised 116,179 weekly records from 526 sites across 42 counties. Data were then aggregated to monthly intervals and spatially filtered using a non-convex hull around sites with at least one EEEV detection, excluding sentinel sites in southern Florida where seroconversion was never recorded (Fig. 1). Records prior to 2005 were removed due to diagnostic limitations between EEEV and Highlands J virus. For each coop, we calculated the monthly proportion of positive chickens (i.e., total number positive in the month / total number tested in the month), weighted by the number tested. Similar to^10^ and general FDOH guidelines, we assumed six chickens were sampled per coop per week, though a gap is present in historical records reporting the exact number of chickens in individual coops each week, which may introduce some uncertainty in derived weights. The dataset was partitioned into training (2005–2017) and testing (2018–2019) subsets, yielding 84,719 monthly records from 476 sites in 39 counties, providing broad coverage of EEEV transmission across Florida.

### Environmental variables

We compiled climate and landscape covariates for each sentinel chicken site. Daymet daily precipitation and minimum and maximum temperatures from 2000 to 2020 at a 1-km^2^ resolution^54^ were downloaded using the ‘climateR’ R package^55^. We then aggregated values to monthly means, before extracting values to site locations using available functions in the ‘terra’ and ‘sf’ R packages^56,57^. To identify temporal lags, we used cross-correlation analyses between statewide monthly EEEV seroconversions and each monthly climate variable at a maximum of 12 monthly lags using the ‘forecast’ R package^58^. Significant lags with coefficients exceeding ± 0.2 identified 1-, 5-, and 12-month lags for precipitation and 1-, 6-, and 12-month lags for both temperature variables.

Land use/land cover (LULC) data were extracted from 30-m^2^ resolution National Land Cover Database data (i.e., 2001, 2004, 2006, 2008, 2011, 2016, 2019)^59^ using the ‘fedData’ R package^60^. Similar to our other work in Florida^10^, we aggregated National Land Cover Data land cover from Anderson Level III classes into three general categories prior to calculating percentages of land cover type surrounding chicken coops. These classes encompass variation across the region, with the goal of identifying generalizable classes that provide predictive signal, as well as transferability for predictions across the region. In this study, our “wetlands” category included woody and emergent herbaceous. We also included a “forest” category, which included only deciduous, evergreen, and mixed forest, and we included a “developed” land cover category with low-, mid-, and high-intensity developed areas. Other land cover classes were not included in our analyses.

We then calculated the proportion of each LULC type within 2.5-km^2^ buffers around sentinel sites. Our choice of buffer distance was based on documented estimates of a flight distance of 2-km^2^ for the primary mosquito vector *Culiseta melanura*, with the potential for further distances based on host seeking behavior^61^. For statewide predictions, we generated 1-km2 environmental covariate grids by computing land cover proportions within 2.5-km^2^ windows and prepared climate variables with corresponding temporal lags at the same 1-km^2^ resolution. All environmental covariates were standardized using z-score normalization based on training data statistics (2005–2017), with scaling parameters applied consistently to testing data (2018–2019) to maintain temporal integrity. For statewide spatial predictions, environmental variables at 1-km^2^ resolution were standardized using training data means and standard deviations.

To assess multicollinearity between environmental predictors, we first calculated binomial generalized linear mixed effects models (GLMMs) with a complementary log–log (cloglog) link function with monthly proportions of EEEV seroconversion weighted by sampling effort as the response variable, landscape and lagged climate variables as predictor variables, and nested site-by-country random intercepts. Predictors were parameterized as second-order polynomials to capture non-linear responses. We then calculated variance inflation factors (VIFs) using a threshold > 5 to identify candidate predictors sets, resulting in 2 candidate sets for subsequent analyses with differing levels of predictor variance inflation (Table S1.5). GLMMs were run in the ‘glmmTMB’ R package and VIFs were calculated using the ‘performance’ R package^62^.

### Model selection

Our overall modeling framework used GLMMs^63^ that explicitly account for spatiotemporal autocorrelation using Gaussian Markov random fields (GMRFs)^64^ approximated using stochastic partial differential equations (SPDE) with Matérn covariance functions and a first order autoregressive (AR1) term^65,66^. For full model specification see Methods S.1. Models were implemented in the ‘sdmTMB’ R package, which combines utility from TMB and INLA^67^.

For computational efficiency, we first performed model selection using spatial binomial GLMMs without a temporal term, using a cloglog link, nested random intercepts for sites within each county, and corresponding environmental predictor variables. Model selection followed a backward stepwise approach, systematically removing predictors and retaining those whose removal most improved Akaike Information Criterion (AIC) scores^68^. To construct spatial meshes required to calculate the SPDE, we followed the approach detailed in^69^ using the ‘fmesher’ R package^70^, using a coarse mesh with 250 vertices to balance model complexity with computational efficiency during the model selection process. We then systematically tested mesh resolutions from 250 to 1,000 vertices by adjusting cutoff parameters (i.e., minimum triangle size), max edge (i.e., maximum triangle edge length), and offset (i.e., spatial extensions) values scaled by factors of the spatial extent of the study area. Model convergence was assessed using ‘sdmTMB’ diagnostic functions, with a 750-vertex mesh (cutoff = 8.5 km), which provided optimal convergence properties without excessive computational costs.

After identifying best performing models from both candidate sets we fitted full spatiotemporal models (Table 1 and Table S1.6), and performance was evaluated using sanity check functions in ‘sdmTMB’ that examined parameter convergence, extreme eigenvalues, standard errors, and random field variances. Residual diagnostics were assessed by generating 500 simulated datasets using maximum likelihood estimates with multivariate normal sampling to assess model adequacy, residual patterns, and potential violations of distributional assumptions, using the ‘DHARMa’ R package^71^.

To determine whether fixed-effect environmental predictors, spatiotemporal structures, and random intercepts improved model performance, we observed marginal AIC values to compare our full spatiotemporal models against seven alternative models including different combinations of these terms (Table S1.1). We calculated conditional percent deviance explained using log-likelihood ratios as *1—(model deviance / null deviance)*, where *deviance* = *− 2* × *log-likelihood* and the null model was the intercept-only non-spatial model. AIC weights were computed using the ‘qpcR’ R package^72^ to quantify relative model support.

Predictive accuracy was evaluated using root mean square error (RMSE) calculations on out-of-sample predictions (2018–2019) and across the full training period (2005–2017) at annual and monthly aggregation levels. Following Thorson and Kristensen (2016)^73^, we applied epsilon bias-correction estimators to obtain accurate temporal predictions of statewide EEEV seroprevalence and associated uncertainty. This approach accounts for bias accumulation when aggregating non-linear model predictions across sites and time periods. We generated 500 simulations using multivariate normal sampling, converted counts to proportions using the binomial size weights, then calculated weighted averages across surveillance sites by month. We then computed temporal indices with multiple confidence intervals (50%, 80%, 95%) to characterize prediction uncertainty over the 15-year time series using the *get_index_sims()* function.

Models predicted monthly EEEV seroprevalence across Florida from 2005 to 2019 using 100 simulations per prediction to quantify uncertainty. Predictions were converted from cloglog link space to the response scale and truncated at various quantiles (90th, 95th, 98th percentiles) for visualization. Annual summaries were derived by averaging monthly predictions, and overall summaries were aggregated across years to map persistent risk. Epsilon bias correction was applied to temporal indices but omitted from full statewide predictions due to computational intensity.

### Avian host–pathogen association analysis

We tested whether there are strong correlations between relative abundance of 12 common bird species that are suspected hosts of EEEV and model-predicted EEEV seroprevalence. In particular, we selected migratory and resident Florida species with differing expected correlations to virus activity, based on prior *Cs. melanura* blood meal analyses in Florida and Alabama^23^. Our migratory species were: American Robin, Eastern Phoebe, Hermit Thrush, Red-eyed Vireo, Wood Thrush, and Yellow-rumped Warbler. Our year-round residents were: Northern Cardinal, Common Yellowthroat, White-eyed Vireo, Green Heron, Pine Warbler, and Black-crowned Night Heron. We obtained weekly species-specific relative abundance estimates from the eBird Status and Trends dataset^74^, modeled as the number of individuals a citizen scientist may observe during a 1-km traveling checklist during the optimal time of day. Raster data are available at 3, 9, and 27-km^2^ resolutions and represent species-specific relative abundance derived across multiple years to represent a “typical year.” To align to the monthly temporal unit of EEEV predictions, we aggregated the weekly relative abundances values to monthly relative abundance values using a standard week-to-month mapping (52 weeks to 12 months). We then processed EEEV model predictions by converting spatiotemporal estimates to monthly rasters at a 1-km^2^ resolution, aggregated to the 3-km^2^ resolution of the eBird data, and next computed mean monthly seroprevalence across the full study period (2005–2019) to derive a single monthly value at each pixel representing predicted virus activity for an average year.

All spatial data were reprojected to UTM Zone 17N (EPSG:32617) and resampled to eBird’s 3-km^2^ resolution using bilinear interpolation, ensuring spatial alignment for correlation analyses. We then calculated pixel-wise Pearson correlations between estimated bird abundance and EEEV seroprevalence for each month, incorporating temporal lags of 0–3 months for migratory species to capture delayed associations between bird migration and EEEV virus activity, and for resident species, only contemporary correlations (0-month lag) were examined due to year-round local populations.

## Supplementary Information

Below is the link to the electronic supplementary material.

Supplementary Material 1

## Data Availability

All code necessary to conduct these analyses are stored in the following Github repository: https://github.com/Campbell-Lab-FMEL/EEEV_forecasting. Georeferenced sentinel chicken seroconversion data is available upon request through the Florida Department of Health Arbovirus Surveillance program upon agreement from participating Florida mosquito control programs through a memorandum of understanding. The authors did not receive special privileges in accessing the data that other researchers would not have. Contact information for data requests are available through the FDOH website: https://www.floridahealth.gov/diseases-and-conditions/mosquito-borne-diseases/surveillance.html.
