# Supplementary material for: Linking hosts, landscapes, and climate to advance zoonotic arbovirus forecasting
Source: Sci Rep. 2026 Apr 6;16:16463. doi: 10.1038/s41598-026-46902-2 (PMC13216555; doi:10.1038/s41598-026-46902-2)
Supplement: Supplementary file 1 — Supplementary Material 1 [file 41598_2026_46902_MOESM1_ESM.pdf]

Supplementary Methods

The spatiotemporal model developed in this study is similar to non-spatial GLMMs but includes additional terms to account for spatiotemporal non-independence. Here, we estimated Gaussian Markov random fields (GMRFs) for spatiotemporal variation ε_t using the SPDE method that approximates a Matérn correlation function.

We evaluated the likelihood for each observation $i \in \{1,2,\ldots,N_{observations}\}$ as proportion data following a binomial distribution, where c_i represents the number of EEEV-seropositive birds out of n_i total birds tested, with $y_i = c_i/n_i$ being the observed proportion and using a complementary log-log link function:

$c_i \sim Binomial(n_i, \mu_i)$,

$\mu_i = 1 - exp(-exp(p_i))$,

$p_i = X_i^T \beta + Z_i^T \alpha + A_i \varepsilon_{t(i)}$,

$\varepsilon_i = \{$

$MVN(0, \Sigma_e)$ if $t = 1$

$MVN(\backslash, \Sigma_e)$ if $t > 1$

$MVN(\rho\varepsilon_{t-1}, (1 - \rho^2)\Sigma_e)$ if $t > 1$

}

where y_i represents the observed proportion of EEEV-seropositive birds at observation i and time t[i]; μ_i represents the true probability of EEEV seropositivity; n_i represents the number of chickens tested (weights = testing × 6 chickens per testing event); X and Z are design matrices for fixed and random effects with coefficient vectors β and α, respectively; ε_t represents the spatiotemporal random field at time t across SPDE vertices; A is the bilinear interpolation matrix such that $A_i\varepsilon_{t(i)}$ interpolates spatiotemporal variation for observation i; Σ_e represents the spatial covariance matrix of the spatiotemporal random field; and ρ represents the first-order

autocorrelation over time in the spatiotemporal term ε_t. The complementary log-log link function relates the linear predictor to the probability μ_i, which is appropriate for modeling rare events and asymmetric probability distributions.

Table S1.1: Description of the model components leading to the development of generalized linear mixed models ("GLMMs") with Gaussian Markov random fields ("GMRF"), including Deviance Explained, marginal AIC and AIC Weight. Models were fitted to seroconversion proportions of Eastern Equine Encephalitis Virus ("EEEV") in Florida at monthly time scales. These models are described in terms of their major components, including intercepts ("~1"), fixed effects, random effects, and Gaussian Markov random fields (GMRF).

Model	Deviance Explained	AIC	ΔAIC	AIC Weight
EEEV ~ GMRF + fixed effects + random effects	0.377	6164.1	0	1
EEEV ~ GMRF + random effects	0.369	6210.2	46	0
EEEV ~ GMRF + fixed effects	0.34	6518.5	354.4	0
EEEV ~ 1 + GMRF	0.331	6573.7	409.5	0
EEEV ~ fixed effects + random effects	0.299	6920.7	756.6	0
EEEV ~ random effects	0.274	7135.5	971.3	0
EEEV ~ 1	0	9822.8	3658.6	0

Table S1.2: Summary of model predictive accuracy when predicting out-of-sample Eastern Equine Encephalitis seroprevalence proportions. Predictive accuracy is measured using root mean square error (RMSE) and reported at the monthly scale with yearly totals, as well as the overall study period (Total) for out-of-sample data for the years 2018 and 2019.

Year	Month	RMSE - Candidate Set 1	RMSE - Candidate Set 2
2018	1	0.00255	0.00255

	2	0.00002	0.00002
	3	0.00305	0.00305
	4	0.00700	0.00700
	5	0.01416	0.01415
	6	0.01172	0.01172
	7	0.01425	0.01425
	8	0.00726	0.00726
	9	0.00318	0.00318
	10	0.00004	0.00003
	11	0.00555	0.00556
	12	0.00255	0.00255
2019	1	0.00360	0.00360
	2	0.00191	0.00191
	3	0.00153	0.00153
	4	0.00360	0.00360
	5	0.01028	0.01028
	6	0.01535	0.01535
	7	0.01349	0.01349

	8	0.00425	0.00425
	9	0.00565	0.00565
	10	0.00270	0.00270
	11	0.00191	0.00191
	12	0.00360	0.00360
Yearly Totals			
2018		0.00767	0.00767
2019		0.00724	0.00724
Total		0.00746	0.00746

Table S1.3: Parameter estimates of predictors from a spatiotemporal model fitted against monthly aggregated EEEV seroprevalence proportions in Florida's sentinel chicken surveillance system. Predictors with Confidence Intervals (95% CI; Lower, Upper) that do not overlap with zero are highlighted in **bold** and are considered important predictors of EEEV seroprevalence.

Variable	Type	Estimate	Std. Error	95% CI (Lower)	95% CI (Upper)
Intercept	*NA*	-10.5257	0.3436	-11.199	-9.8523
Precipitation Lag 1 Month	*Linear*	10.831	19.0896	-26.5838	48.2459
Precipitation Lag 1 Month	*Quadratic*	-3.2013	15.3243	-33.2363	26.8338

Precipitation Lag 5 Months	*Linear*	-18.8877	20.4343	-58.9382	21.1628
Precipitation Lag 5 Months	*Quadratic*	-24.9001	19.0994	-62.3343	12.5341
Precipitation Lag 12 Months	*Linear*	36.3576	18.3542	**0.384**	**72.3312**
Precipitation Lag 12 Months	*Quadratic*	-30.315	14.1791	**-58.1056**	**-2.5244**
Tmax Lag 6 Months	*Linear*	-37.9701	56.2275	-148.174	72.2337
Tmax Lag 6 Months	*Quadratic*	38.4038	21.9422	-4.6022	81.4097
Tmax Lag 12 Months	*Linear*	-9.8866	54.723	-117.142	97.3686
Tmax Lag 12 Months	*Quadratic*	11.7183	26.0875	-39.4122	62.8489
Tmin Lag 1 Month	*Linear*	76.811	40.4647	-2.4983	156.1204
Tmin Lag 1 Month	*Quadratic*	-67.3957	28.0519	**-122.376**	**-12.4151**
Forest Cover	*Linear*	142.3936	45.2068	**53.7899**	**230.9973**
Forest Cover	*Quadratic*	-87.0073	30.0387	**-145.882**	**-28.1325**

Wetland Cover	*Linear*	128.5891	32.5892	**64.7155**	**192.4627**
Wetland Cover	*Quadratic*	-44.5205	30.6417	-104.577	15.5361

Table S1.4: Parameter estimates of predictors from a spatiotemporal model fitted against monthly aggregated EEEV seroprevalence proportions in Florida's sentinel chicken surveillance system. The variables included in this model were those that were removed based on initial VIF screening and fitted as an alternative candidate set. Predictors with Confidence Intervals (95% CI; Lower, Upper) that do not overlap with zero are highlighted in **bold** and are considered important predictors of EEEV seroprevalence.

Variable	Type	Estimate	Std. Error	95% CI (Lower)	95% CI (Upper)
Intercept	*NA*	-10.5292	0.346	-11.2075	-9.851
Tmin (Current)	*Linear*	136.385	66.3949	6.2534	266.5165
Tmin (Current)	*Quadratic*	22.1909	34.736	-45.8903	90.2721
Tmin Lag 6 Months	*Linear*	-59.6911	57.672	-172.726	53.3439
Tmin Lag 6 Months	*Quadratic*	22.9416	20.5329	-17.3022	63.1853
Tmin Lag 12 Months	*Linear*	-99.4721	70.6447	-237.933	38.9891
Tmin Lag 12 Months	*Quadratic*	61.264	34.09	-5.5512	128.0792

Tmax Lag 1 Month	*Linear*	46.5541	51.4576	-54.301	147.4091
Tmax Lag 1 Month	*Quadratic*	-96.7682	30.8724	**-157.277**	**-36.2595**
Forest Cover	*Linear*	143.511	45.6413	**54.0557**	**232.9663**
Forest Cover	*Quadratic*	-86.9773	30.1115	**-145.995**	**-27.9599**
Wetland Cover	*Linear*	129.2191	32.5968	**65.3305**	**193.1077**
Wetland Cover	*Quadratic*	-44.8197	30.6592	-104.911	15.2712

Figure S1.1: Statewide predictions of the spatiotemporal dynamics of EEEV seroprevalence from the Florida sentinel chicken system during the years of 2001 – 2019. Predictions were obtained from a spatiotemporal model fitted to data aggregated to monthly proportions of EEEV seroprevalence in a sentinel chicken system. All predictions are provided with a quantile truncation for ease of visualization (98th percentile). Maps were created using ‘ggplot2’ package in R version 4.5.2.

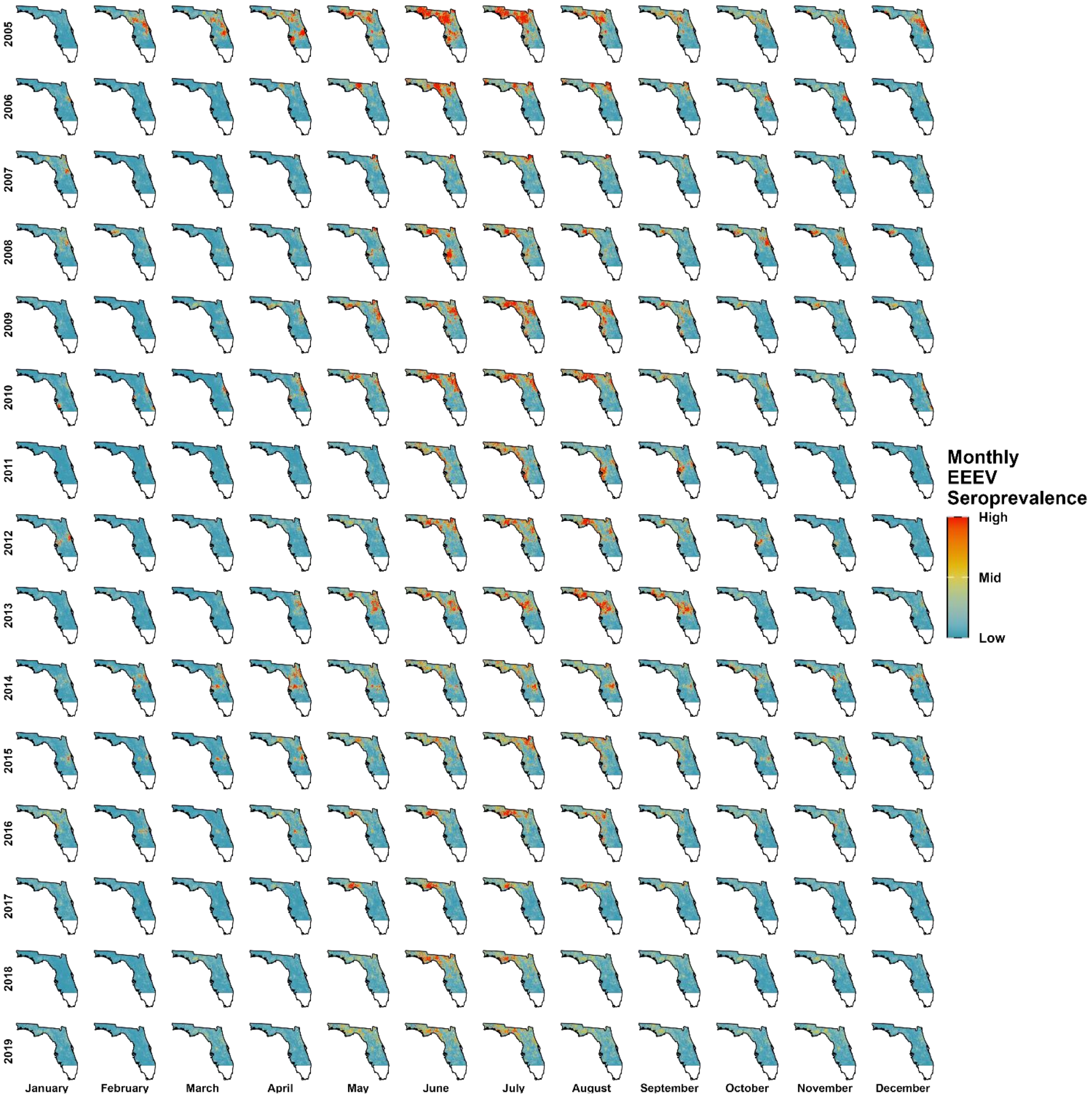
2005
2006
2007
2008
2009
2010
2011
2012
2013
2014
2015
2016
2017
2018
2019
January
February
March
April
May
June
July
August
September
October
November
December
Monthly
EEEV
Seroprevalence
High
Mid
Low

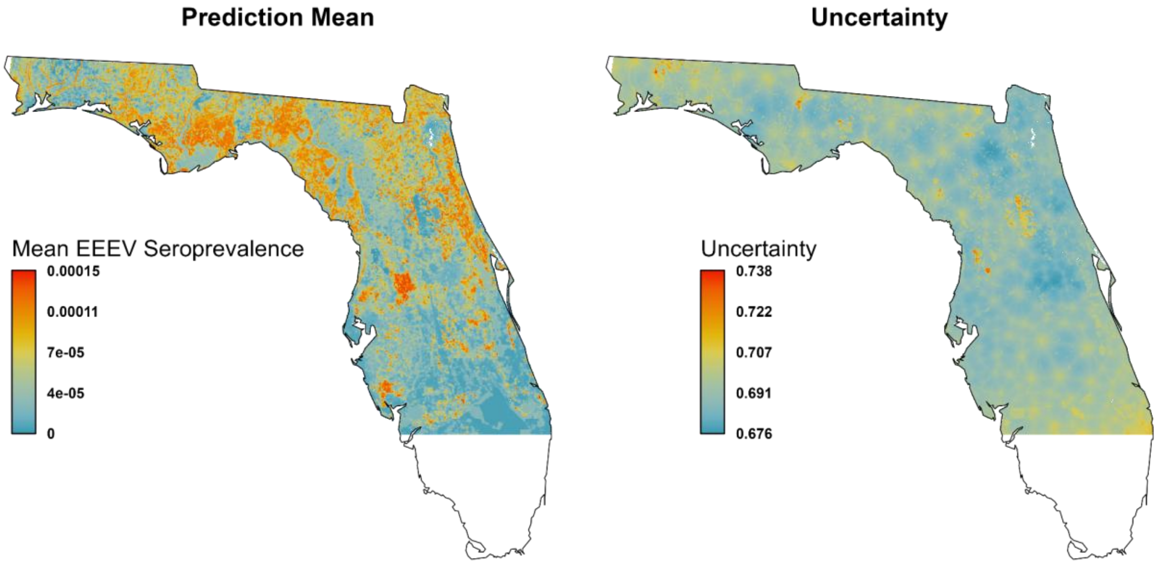

Figure S1.2: Summary of statewide prediction means and associated uncertainty of EEEV seroprevalence during the years of 2005 - 2019 in the Florida Sentinel Chicken Surveillance system. Predictions were obtained from a spatiotemporal model fitted against monthly aggregated seroprevalence proportions. Predictions are mapped using 98th percentile quantile truncation for ease of visualization. Maps were created using ‘ggplot2’ package in R version 4.5.2.

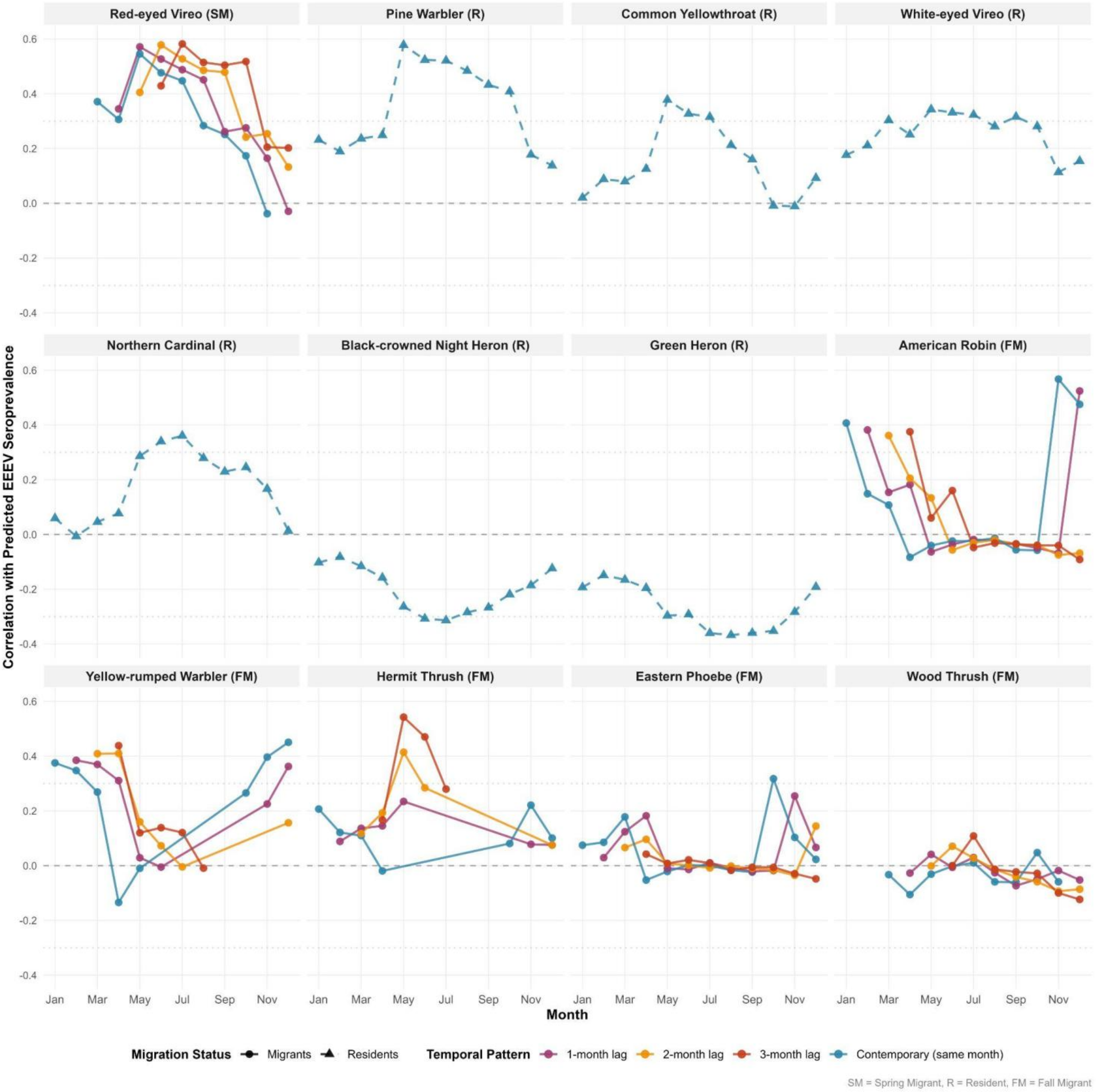

Figure S1.3: Pixelwise Pearson correlations between predicted monthly EEEV seroprevalence and monthly bird abundance across Florida. EEEV predictions were temporally averaged across the study period, while weekly eBird abundance estimates were aggregated to monthly resolution. For resident species, we assessed contemporaneous correlations only. For migratory species, we evaluated both contemporaneous correlations and temporal lags of 1-3 months to account for delayed associations following migration events.

Table S1.5: Results from backward stepwise variable selection to identify the best predictors of monthly Eastern Equine Encephalitis Virus (EEEV) seroprevalence. We first removed highly collinear variables to create two candidate sets, then systematically dropped the least important variables based on AIC improvement. All models included nested random effects (sites within counties) and were weighted by monthly testing frequency. All predictors were modeled as quadratic terms.

Candidate Set	Step	Variable Removed	AIC	ΔAIC	Remaining Predictors
1	0	(starting model)	6930.3	-	precipitation (current + 1,5,12 month lags), temperature max (current + 6,12 month lags), temperature min (1 month lag), developed land, forest, wetlands
	1	developed cover	6927.8	2.5	precipitation (current + lags), temperature max (6,12 month lags), temperature min (1 month lag), forest, wetlands
	2	current temperature max	6925.6	2.2	precipitation (current + lags), temperature max (6,12 month lags), temperature min (1 month lag), forest, wetlands
	3	current precipitation	6924.7	0.9	precipitation (1,5,12 month lags), temperature max (6,12 month lags), temperature min (1 month lag), forest, wetlands
2	0	(starting model)	6879.4	-	temperature min (current + 6,12 month lags), temperature max (1 month lag), developed land, forest, wetlands
	1	developed cover	6877.9	1.5	temperature min (current + 6,12 month lags), temperature max (1 month lag), forest, wetlands

Table S1.6: Description of the predictor variables used to model spatiotemporal EEEV seroprevalence in the state of Florida (USA). Terms included in this table were those that were removed during initial VIF screening of variables and fit as an alternative candidate set. Descriptions and parameterization details are provided for each variable.

Effect	Term	Description	Variable type	Parameterization
Fixed	*tmin*	Min temperature	Continuous	2nd order orthogonal polynomial
	tmin_lag6	Min temperature (6 mo. lag)	Continuous	2nd order orthogonal polynomial
	tmin_lag12	Min temperature (12 mo. lag)	Continuous	2nd order orthogonal polynomial
	tmax_lag1	Max temperature (1 mo. lag)	Continuous	2nd order orthogonal polynomial
	forest	Proportion forest cover	Continuous	2nd order orthogonal polynomial
	wetlands	Proportion wetlands	Continuous	2nd order orthogonal polynomial
Random	*county*	County name	39-level factor	Intercept nested above site_id

	site_id	Monitoring site name	476-level factor	Intercept nested below county
